# Bacterial Fimbriae Designed to Stay with the Flow

**DOI:** 10.1371/journal.pbio.0040314

**Published:** 2006-08-29

**Authors:** Liza Gross

The human digestive system houses a diverse colony of beneficial bacteria, but one species—*E. coli*—can wreak havoc when it colonizes mucous membranes that normally exist unmolested (for example, in the urinary tract). To latch on to cells and establish infection, *E. coli* uses fimbriae—long, hairlike organelles that project from the bacterium’s surface. Fimbriae consist of interlinking subunits of a single protein called pilin that forms a rigid, coiled helix-shaped rod. Sticky proteins called adhesins cap the tip of the rod and bind to carbohydrate receptors on their host, thus securing bacteria on the host cells as extracellular fluids swirl around them.

A previous study led by Evgeni Sokurenko and Viola Vogel investigated the most common type of *E. coli* fimbriae. The sticky protein at the tip of these fimbriae is called FimH and binds to a carbohydrate called mannose. They showed that powerful drag forces created by the extracellular fluids don’t carry the bound bacteria away, as one might expect, but instead strengthen their adhesion to their host. The researchers attributed this increased binding to a biphasic “catch bond” mechanism whereby increased drag forces cause the FimH at the tip of the fimbria to switch from a form that binds mannose weakly to a form that binds strongly. Because of this, the bacteria bind best at an optimal force that is high enough to switch FimH to strong binding but not so high that it breaks the strong FimH–mannose bond.

And now, in a new study, the same group of researchers (including first author Manu Forero) set out to determine whether the coiled rod structure of fimbriae affects how the sticky FimH at the tip binds. It had been assumed that fimbrial rods play a largely static structural role, either by extending the tip adhesins’ reach or by resisting electrostatic repulsive forces between bacteria and cell surfaces. But Forero et al. show that the rods function more dynamically, using their mechanical properties to help stabilize the FimH–mannose bond against a turbulent background.

Fimbriae-mediated adhesion was investigated with an atomic force microscope, which uses a cantilever to apply (and measure) forces between its tip and the sample under investigation. Forero et al. outfitted the cantilever tip with mannose, and then used this to touch a fimbriated *E. coli* cell that was affixed to a glass surface. After mannose bound to the fimbrial FimH, the cantilever retreated from the bacterium at a constant velocity. The researchers determined that, instead of the FimH–mannose bond breaking, the fimbriae stretched out far beyond their original length.

**Figure:**
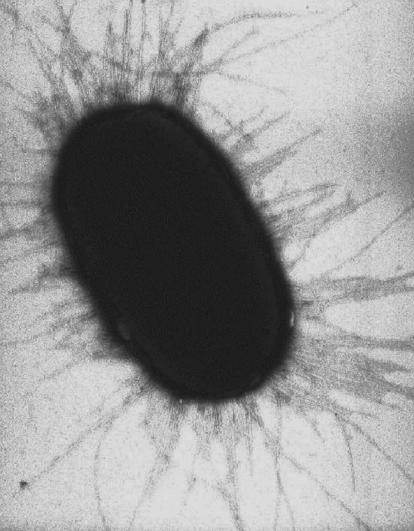
The long, sticky filaments covering *E. coli* bacteria uncoil under force, apparently improving the binding of the terminal adhesive unit in the presence of forces generated by fluid flow. (Image: Manu Forero)

One reason that fimbriae extend could be that the individual pilin subunits of the fimbrial rod are uncoiling. The researchers tested this hypothesis by applying a constant force between the cantilever and fimbria—under which fimbrial length changes slowly. They observed that stepwise jumps in distance corresponded to the expected length of individual subunits unwinding from the coiled shaft one at a time. Thus, the researchers concluded, fimbrial elongation proceeds as subunits uncoil one after another. This was also supported by a mathematical model developed by the researchers to quantify the biophysical forces governing the dynamics of fimbrial uncoiling.

Forero et al. also detected that, after uncoiling at increasing force, the stretched fimbriae re-coil if the pulling force drops. Importantly, while fimbrial uncoiling under high force decreases the tension within the rod, re-coiling under low force increases the tension. Thus, the tensile force within the rod stays within some intermediate level when fimbrial length is stable.

The researchers found that the intermediate force range corresponds to the force level where the FimH–mannose bonds last longest. Lower, coiling forces are too weak to switch bonds to a long-lived state before breaking, and higher, uncoiling forces exceed the catch-bond threshold, shortening the life of the bond. Because *E. coli* living in the gut or other mucosal surfaces experience constantly changing flow rates and forces, these adjustments should enhance fimbrial attachment under a diverse range of fluid conditions. The correspondence of forces suggests that the mechanical properties of the fimbrial rod and the FimH–mannose complex co-evolved to optimize adhesive stability in fluids.

